# In Vitro Evaluation of Bioavailability of Mg from Daily Food Rations, Dietary Supplements and Medicinal Products from the Polish Market

**DOI:** 10.3390/nu17050748

**Published:** 2025-02-20

**Authors:** Piotr Bawiec, Agnieszka Jaworowska, Jan Sawicki, Marcin Czop, Radosław Szalak, Wojciech Koch

**Affiliations:** 1 Department of Food and Nutrition, Medical University of Lublin, 4a Chodźki Str., 20-093 Lublin, Poland; piotr.bawiec@wp.pl (P.B.); agnieszka.jaworowska@umlub.pl (A.J.); radek.szalak@up.lublin.pl (R.S.); 2 Department of Analytical Chemistry, Medical University of Lublin, 4a Chodźki Str., 20-093 Lublin, Poland; jansawicki@umlub.pl; 3 Department of Clinical Genetics, Medical University of Lublin, Radziwiłłowska 11 Str., 20-080 Lublin, Poland; marcin.czop@umlub.pl; 4 Department of Animal Anatomy and Histology, Faculty of Veterinary Medicine, University of Life Sciences, 12 Akademicka St., 20-950 Lublin, Poland

**Keywords:** diets, bioavailability, ICP-OES, trace elements

## Abstract

Background: Nutrients consumed with food undergo the digestion process, and only some of them are absorbed from the gastrointestinal tract (GI) and enter the bloodstream. Minerals, including Mg, are crucial for maintaining the body’s homeostasis, but their beneficial effect depends on their bioavailability, i.e., the part that can be absorbed and used by the body. The bioavailability of nutrients taken in pharmaceutical form is usually higher than the same nutrients contained in food, because their absorption requires prior release from the food matrix. Objectives: The main objective of the conducted research was to assess the bioavailability of Mg from dietary supplements and medicinal products, considering the influence of the type of diet and pharmaceutical form on bioavailability. Methods: The experiments were conducted using a previously developed and optimized two-stage in vitro digestion model using cellulose dialysis tubes and ICP-OES method. The influence of three types of diets—standard, basic and high-residue—on the bioavailability of Mg was evaluated. Results: The bioavailability of Mg from the studied diets was within the range of 48.74–52.51%. Conclusions: In the models studied, it was observed that the factors influencing bioavailability were the nutritional composition of the diets and the chemical form of Mg.

## 1. Introduction

Magnesium (Mg) is in fourth place (after calcium, sodium, and potassium) among the mineral components that make up the human body. Moreover, it is the second most abundant intracellular cation, after potassium [1,2]. In the body of an adult weighing 70 kg, the average magnesium content is 25 g. The majority of this element, as much as 53%, is stored in the bones, 27% serves as a reserve in the muscles, and 19% is part of parenchymal organs and soft tissues. Only about 1% of magnesium is present in the blood serum [2,3]. Magnesium levels in the body are controlled by three main mechanisms: absorption in the intestines, absorption and excretion processes in the kidneys, and exchange with internal reserves, located mainly in the bones. The intestines, bones, and kidneys play a key role in maintaining magnesium homeostasis. About 30–50% of magnesium is absorbed in the distal small intestine and colon, while the bones act as the largest storage site for this element. The kidneys, on the other hand, are responsible for regulating its excretion, which allows for the precise adjustment of magnesium levels in the body [1,4]. According to the European Food Safety Authority (EFSA), the reference daily intake value for Mg is 300 mg for women and 350 mg for men. Higher Mg requirements may be needed in some physiological conditions, such as pregnancy, aging or during intense physical activity, as well as in some pathological conditions, such as infections or type 2 diabetes [5,6]. The primary source of various nutrients, including trace elements such as magnesium, is the food of plant and animal origin, which must be regularly supplied to the body [7,8]. The importance of minerals (macro- and microelements) for human health depends not only on their quantity consumed but also on the fraction that can be absorbed and then used or stored in the body [9,10]. Analytical methods that focus on assessing the content of various elements in food and their dietary intake play a key role in determining their concentrations in various food products and in accurately determining reliable consumption levels. This allows for a better understanding of their importance in the diet and their impact on human health. A key element of such studies is also the analysis of bioavailability, which indicates the actual amount of a substance that reaches the bloodstream and can produce an effect in the body [9,10,11,12,13]. Bioavailability can be defined as a process involving the following stages: release of the ingredient from the organic matrix in the gastrointestinal tract, its transition through the intestinal wall into the body and metabolism, including decomposition in the intestines and liver, ending with the introduction of the ingredient into the systemic circulation. The bioavailability of nutrients taken orally in a pure form is usually higher than that of the same nutrients contained in a food matrix because absorption requires their prior release [13,14]. The assessment of the digestibility of dietary components can be conducted by determining the in vivo digestibility parameters, i.e., using a human or animal model, or in vitro digestibility by determining the degree of enzymatic hydrolysis of these products in laboratory conditions [15]. On the one hand, in vitro digestion systems are a good choice for preliminary, fast and cheap assessment of the bioavailability of elements from a diet or pharmaceutical preparations. However, to go further, to assess to what extent it will be absorbed into the bloodstream of the host, advanced analytical methods are needed, which are currently too demanding for routine use in the laboratory, so further development work is necessary [16]. Therefore, a mixed method, which combines in vitro studies using digestive enzymes, appropriate pH and temperature, extended by the use of dialysis tubes, which mimic intestinal absorption, is often considered a compromise solution between in vitro and in vivo methods and is considered to be an appropriate model in the study of the bioavailability of mineral components from food, supplements and drugs [17,18,19].

The main objective of the conducted studies was to assess the influence of various types of diets on the bioavailability of Mg from dietary supplements and medicinal products. The studies were conducted using a two-phase in vitro digestion model and cellulose dialysis tubes. Additionally, the influence of the chemical forms of the element and the pharmaceutical forms of the studied preparations on the effectiveness of their absorption was also analyzed. Because a well-balanced diet should contain food products from all nutritional groups, the studies used reconstructed daily food rations (DFR) in the form of homogenates consisting of four meals. To better analyze the assessment of mutual dependencies, several preliminary studies were performed to assess the degree of bioavailability of the preparations selected for the studies.

To date, many studies have been conducted to assess the bioavailability of Mg from supplements and drugs, focusing mainly on the differences between organic and inorganic compounds. However, to the best of our knowledge, the present study is the first to thoroughly analyze the effect of diet type and food matrix composition on the bioavailability of this element using in vitro model with dialysis tubes and advanced analytical methods.

## 2. Materials and Methods

### 2.1. Chemicals and Reagents

HNO_3_, HCl, and H_2_O_2_ used during determinations were of Suprapur grade and were bought from Merck (Darmstadt, Germany). Mg standard solution (50 mg/L) was purchased from PlasmaCAL (SCP Science, Baie-D’Urfé, QC, Canada). Enzymes used during the in vitro digestion stage (pepsin and pancreatin) and dialysis tubes with a molecular weight cut-off (MWCO) of 14 kDa were purchased from Sigma-Aldrich (St. Louis, MO, USA). Sodium bicarbonate was supplied by Avantor Performance Materials (POCH, Gliwice, Poland). High-purity deionized water (resistivity 18.2 MΩcm) obtained using an Ultrapure Millipore Direct-Q-R 3UV (Millipore, Bedford, MA, USA) was used in all analytical steps. SCP Science, Canada, was the supplier of all certified laboratory equipment used during trace elements determinations (DigiTubes and DigiFilters).

### 2.2. Materials

#### 2.2.1. Dietary Supplements and Medicinal Products

A total of 12 products officially approved in Poland were used in the study, of which 9 were registered as dietary supplements and 3 were medicinal products. It is worth noting that, unlike dietary supplements, medicinal product packaging does not provide the reference intake value (% RDA), which is standardly indicated on supplements. The research material was selected based on the popularity of Mg chemical compounds chosen by consumers in the largest pharmacy chains in Poland. The inclusion criteria included the following: dietary supplements from the “DS” product category, wide availability on the pharmaceutical market and products with a valid expiration date, as well as drugs containing Mg from the “OTC” category (products available over the counter). Exclusion criteria included difficult accessibility during the study. Trade names of drugs and dietary supplements were replaced with numbers because the major goal of the study was to evaluate the bioavailability of Mg in various chemical forms and their quality or the quality of their manufacturers. Detailed characteristics of the supplements and drugs analyzed in the study are presented in Table 1.

This study was conducted in 2021–2022. Three samples of each product from three different batches were analyzed. All analyses were performed in triplicate. Mg bioavailability was assessed in 39 different experimental models (36 models using dietary supplements or medicinal products and 3 models for diets). Taking into account the number of dietary supplements and drugs, the types of diets used, and the number of replicates, a total of 336 samples were analyzed (36 models × 3 different batches × 3 replicates and 3 types of diets × 3 replicates and 3 control samples). Considering that according to the procedure used in the current study, after each in vitro digestion, two fractions were obtained, the concentration of Mg was determined in a total number of 672 analytical samples. Additionally, Mg concentration was determined in 6 samples of the reference material. Therefore, a total number of 678 analytical samples was subjected to ICP-OES determinations.

#### 2.2.2. Reconstructed Diet Duplicates

To determine the bioavailability of Mg in the context of different dietary patterns, three types of diets most commonly used in the nutrition of healthy people were developed. These patterns were based on literature data from the field of dietetics and the practical knowledge of professional dietitians [20,21,22]. Nutritional models were developed and applied in the experimental part of this study. Three types of diet were developed and reconstructed: standard, basic and high-residue (also called high-fiber diet). Dieta 6.0 software [National Institute of Public Health–National Institute of Hygiene, Warsaw, Poland] was used to calculate the nutritional values of each diet. Tables S1 and S2 in the Supplementary Materials present a detailed information on the composition and nutritional values of the reconstructed diets (DFR). A detailed description of the process of preparing and reproducing DFRs has been previously presented [23,24]. All products used to prepare the diets came from the retail market of the Lublin region, and over 90% of them were of local origin. The meals included in the diets were prepared in the laboratory in accordance with the guidelines contained in the literature and using a previously tested and approved procedure [11,24,25,26,27]. Stainless steel equipment was used for food preparation to avoid food contamination. Additionally, all laboratory equipment was thoroughly cleaned after each use using laboratory-grade detergent, hot tap water, low-concentration Suprapur-grade acid solution, and ultrapure deionized water. Diets were stored in plastic containers, and homogenization equipment was thoroughly cleaned before each use to prevent the risk of contamination. Reconstituted diets were homogenized and then stored at −20 °C before analysis.

### 2.3. Two-Phase Enzymatic Model of In Vitro Digestion

This study used a modified in vitro digestion model developed by Miller’s team, one of the first models of digestion in the laboratory. The model scheme includes two stages of digestion—gastric and intestinal—using digestive enzymes, appropriate temperature and pH that allow enzyme activity. The method was improved by using cellulose dialysis tubes, which better mimic the natural conditions in the gastrointestinal tract [18,28]. The procedure used in this study was optimized and previously verified and validated for its accuracy in assessing the bioavailability of various food components, including trace elements, as previously described [23,24,29].

Before starting the first stage of digestion, 25 g of diet homogenate was weighed into special tightly closed containers made of polypropylene (PP) laboratory material and filled up with ultra-pure water to 50 g. In models with dietary supplements or medicinal products with Mg, the situation was analogous. An amount of 25 g of diet homogenate was weighed and mixed with 1 portion of pharmaceutical product corresponding to one tablet, coated tablet, capsule or sachet, and the entire system was topped up with ultra-pure water to a final mass of 50 g. Models in which no pharmaceutical products were added were created to assess the bioavailability of Mg from diet alone. At the beginning, before the whole procedure started, the pH of the digestion system was reduced with 2 mol/L HCl to the level value 2. After reaching the desired pH, 2 mL of a 10% solution of pepsin in 0.1 mol/L HCl was added to each sample. The samples prepared in this way were tightly closed and placed for 2 h in a thermostatic water bath with a shaker (Vibra, AJL electronic, Krakow, Poland) at 37 °C.

After completing this stage, the pH of the sample solution was raised from 2 to 6.5 using a 6% NaHCO_3_ solution. When the target pH value was reached, 5 mL of 0.4% pancreatin solution in 0.1 mol/dm^3^ NaHCO_3_ was added to each sample. In the next digestion step (intestinal digestion), after the pH update, samples were transferred to cellulose dialysis tubes, which had been previously soaked for 12 h in 0.1 mol/L HCl and rinsed several times with ultra-pure water. Dialysis tubes were hermetically sealed, placed in PP containers containing 500 mL of ultra-pure water in a position where its content was completely immersed in water. Containers were sealed and shaken for 2 h in a special laboratory shaker, at a constant temperature of 37 °C during the experiment, as it was previously described. After this step, two fractions were obtained—the dialysate, and the residues remaining inside the tubes. These both fractions were subjected to further determinations–digestion procedure and elemental analysis using the ICP-OES method.

**Figure 1:**
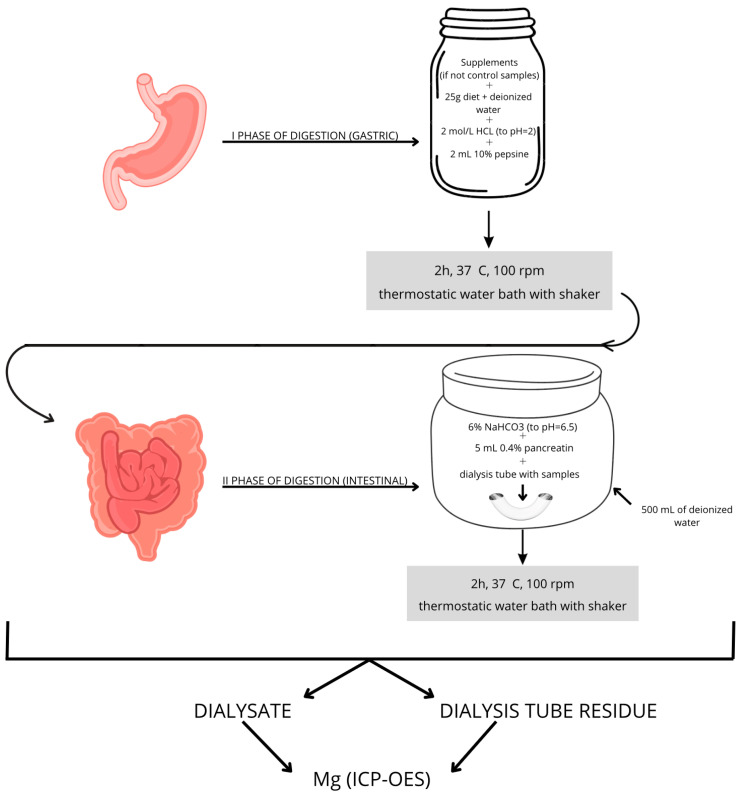
The scheme of the experimental procedure.

At the same time, and under the same conditions, control samples were analyzed, which were treated identically to the research samples, with the difference that no analyte (dietary supplement, medicinal product, or dietary components) was inside the reaction mixture. Graphical illustration of the conducted experiment was presented in Figure 1. 

### 2.4. Analytical Determination of Mg

DigiPREP MS (SCP Science, Baie-D’Urfé, QC, Canada) with a condensate recirculation system was used for sample digestion. Dialysate solutions (5 mL) were digested with 1 mL of concentrated (65%) HNO_3_ for 120 min at 120 °C. The residues in the dialysis tubes were digested with a mixture of 65% HNO_3_ and 30% H_2_O_2_ for 120 min at the same temperature. The obtained digest solutions, after cooling, were filtered using a Rocker 300 vacuum pump (Rocker Scientific, New Taipei City, Taiwan) and DigiFILTER filters. Then, the samples were diluted with ultra-pure water to a final volume of 10 mL. The Mg concentration in the samples was determined by high-resolution ICP-OES (inductively coupled plasma optical emission spectrometry) using a PlasmaQuant 9000 Elite spectrometer (Analytik Jena, Jena, Germany). All analyses were performed in triplicate, and the details of instrumental settings are described in Table S3 in the Supplementary Materials. The calibration of the equipment was performed using standard Mg PlasmaCAL solutions (SCP Science, Baie-D’Urfé, QC, Canada) (50,000 μg/mL), appropriately diluted. The applied analytical protocol, previously verified for its accuracy and precision in the determination of trace elements, including Mg [26,30], was re-tested considering other digestion protocol. For this purpose, a mixture of flour and milk powder (in a ratio of 7:3 *w*/*w*) was used, enriched with a specified amount of different elements, including Mg. All analytical samples (including the reference material) were analyzed simultaneously under the same conditions. The results were presented in Table 2.

### 2.5. Calculation of the Bioavailability Value

Based on analytical determinations the value of Mg bioavailability was calculated using equations presented below, as previously described [23,24].

$$B\%=\frac{D+Dr}{T+D\times 100\%}$$

where B% is the bioavailability of Mg (in %), D and T refer to the amount of Mg (in mg) in the dialysate and the residue inside the dialysis tube, respectively. Dr represents the concentration of Mg (in mg), which corresponds to the equilibrium concentration on both sides of the cellulose membrane inside the dialysis tube. Dr was calculated using the following formula:

$$\mathrm{Dr}=\frac{\left(Cd-Ck\right)\times Vt\times R}{1000}$$

where C_d_ is the Mg concentration in the dialysate solution (in μg/mL), C_k_ is the Mg concentration in the control sample (in μg/mL), V_t_ is the volume of the dialysis tube (in mL), and R is the dilution factor.

### 2.6. Statistical Analysis

Statistica software version 13.0 (StatSoft, Krakow, Poland) and MS Excel 2010 (Microsoft, Washington, DC, USA) were used for statistical calculations. Descriptive statistics methods were used to present the results, such as the arithmetic mean (x, M), median (Me), standard deviation (SD), interquartile range (IQR), minimum (Min) and maximum (Max) values, as well as test statistics (F) and (H) for the ANOVA test, which allowed for the analysis of quantitative data. The Shapiro–Wilk test was used to assess the distribution of the obtained results. Parametric and nonparametric tests were used, for data with a normal and non-normal distribution, respectively.

To evaluate the differences between the bioavailability of Mg in particular models, one-way analysis of variance (ANOVA) with Tukey’s post hoc test was used. When evaluating the effect of chemical compounds of Mg in supplements particular products or their pharmaceutical forms on the value of bioavailability, one-way ANOVA analysis with Tukey’s post hoc test and Kruskal–Wallis rank test with Dunn’s post hoc test were used.

The critical level of significance (α) in all tests was assumed to be 0.05 (α = 0.05). The following significance levels were adopted in the study: *p* < 0.05—statistical significance, *p* < 0.01—strong statistical significance, *p* < 0.001—very strong statistical significance.

## 3. Results

### 3.1. Bioavailability of Mg Under the Influence of Various Diets

The major goal of this study was to determine the effect of different types of diets (standard, basic and high-residue) on the bioavailability of Mg from selected dietary supplements and medicinal products. For this purpose, a two-phase in vitro digestion model and chemical analyses were used to determine the element concentration in the obtained fractions. In addition, an assessment of the effect of the chemical and pharmaceutical forms of supplements and drugs on the value of the bioavailability of the studied element was included. The results of Mg bioavailability from the tested dietary supplements and medicines considering the type of the diet used were presented in Table 3. Additionally, the data obtained in the model without the use of pharmaceutical products and dietary supplements illustrate the bioavailability of the element resulting only from a specific type of diet.

The bioavailability of Mg from the studied diets was within the range of 48.74–52.51%. Standard diet was characterized by the highest bioavailability of Mg (52.51%), whereas the lowest results were obtained for the high-fiber diet (48.74%); however, the differences were small and insignificant.

In experiments using dietary supplement No. 1, the obtained results were within the range of 44.95–74.20%. A statistically significant lower bioavailability of Mg from the standard diet was demonstrated compared to the basic (*p* < 0.001) and high-fiber diet (*p* < 0.001). The values for basic and high-fiber diets were very similar and the difference between them was insignificant.

In turn, studies using dietary supplement No. 2 showed a statistically significant lower bioavailability of Mg from the standard diet compared to the basic (*p* < 0.001) and high-fiber diet (*p* < 0.001). Differences between other models were insignificant. For this dietary supplement, the results were within the range of 55.95–66.01%.

Further, in studies using medicinal product No. 3 the results ranged from 44.95 to 74.20%, and a statistically significant higher bioavailability of Mg from the standard diet was demonstrated compared to the basic diet (*p* < 0.001) and high-fiber diet (*p* < 0.001). Differences between the values for basic and high-fiber diets were insignificant.

For dietary supplement No. 4, the obtained results ranged from 69.29% to 70.51%. No statistically significant differences between particular diets were shown.

On the other hand, for dietary supplement No. 5, the averaged results of the bioavailability of Mg under the influence of the diets used in the study ranged from 36.54% to 64.79%. Statistically significant differences were demonstrated between the values for all diets. A lower bioavailability of Mg was observed from the standard diet compared to the basic (*p* < 0.001) and the high-fiber diet (*p* < 0.001). Additionally, significant higher results in a model using the basic diet compared to the high-fiber diet were also demonstrated (*p* < 0.001).

Obtained results for dietary supplement No. 6 ranged from 59.60% to 76.55%, and the results between various models were significant. The highest results were observed for the model in which the standard diet was used compared to the basic (*p* < 0.001) and high-fiber diets (*p* < 0.001). Moreover, significantly higher bioavailability of Mg in the presence of the basic diet compared to the high-fiber diet (*p* < 0.05) was also demonstrated.

Results from 42.06% to 73.03% were obtained for dietary supplement No. 7. In experiments using this dietary supplement, a statistically significant lower bioavailability of Mg in model using the standard diet was revealed compared to the basic (*p* < 0.001) and the high-fiber diet (*p* < 0.001). Between basic and high-fiber diets, the differences were insignificant.

Studies using dietary supplement No. 8 revealed a statistically significant higher bioavailability of Mg in the presence of the basic diet (70.08%) compared to the standard (65.15%) (*p* < 0.01) and high-residue diet (64.76%) (*p* < 0.01). For other models, the differences were insignificant.

In the case of medicinal product No. 9, the average results of the bioavailability of Mg in the presence of various types of diets ranged from 53.50% to 61.42%. Statistically significantly higher results were obtained for a high-fiber diet compared to a standard (*p* < 0.001) and a basic diet (*p* < 0.05). Between other models, no significant differences were revealed.

Results of the bioavailability of Mg in experiments in a model with dietary supplement No. 10 ranged from 52.86% to 61.80%. A statistically significant lower bioavailability of Mg was in the presence of the standard diet compared to the basic diet (*p* < 0.001). A statistically significant lower results were also demonstrated in the case of the standard diet compared to the high-fiber diet (*p* < 0.001) and in the case of the basic diet compared to the high-fiber diet (*p* < 0.01).

On the other hand, in the case of the use of medicinal product No. 11, the average results ranged from 60.65% to 75.27%. A lower bioavailability of Mg was observed from the standard compared to the basic (*p* < 0.001) and the high-fiber diet (*p* < 0.001). In experiments using this medicinal product, the highest results were determined for the basic diet, which were significantly higher than for the high-fiber diet (*p* < 0.001).

Research on the latest supplement number 12 has revealed, that the average results ranged from 50.09% to 58.54%. In experiments using this dietary supplement, a statistically significant higher bioavailability of Mg in model in which the basic diet was used was demonstrated compared to the standard (*p* < 0.05) and the high-fiber diet (*p* < 0.001). Results between the standard and high-fiber diets were insignificant.

### 3.2. Influence of Diet and Chemical Form on the Bioavailability of Mg

Table 4 and Table 5 present the bioavailability of Mg under the influence of the studied diets, considering the chemical forms of the element. Table 4 presents a detailed analysis considering the type of the diet, whereas in Table 5 only the average results for particular Mg compounds were presented. Mg, in the dietary supplements and medicinal products used during the experiments, occurred in seven chemical forms: magnesium hydroxide, magnesium oxide, magnesium bisglycinate (ALBION^®^ magnesium amino acid chelate), magnesium lactate dihydrate, magnesium citrate, magnesium chloride hexahydrate and magnesium L-pidolate. Each chemical form was analyzed for its effect on the bioavailability of Mg in the presence of each diet used in the study.

In models in which magnesium chloride hexahydrate was used, the results of the bioavailability of Mg were the highest (68.37%). On average, a statistically significant higher bioavailability of Mg in the presence of the studied diets was demonstrated in experiments using the chemical form of magnesium hydroxide (59.76%), compared to magnesium lactate dihydrate (57.12%) (*p* < 0.01), magnesium citrate (57.53%) (*p* < 0.05), magnesium L-pidolate (53.86%) (*p* < 0.001) and models in which no dietary supplement or medicinal product (51.16%) (*p* < 0.001) were used, which refer to a simulated bioavailability from a natural, mixed diet. However, the results for magnesium oxide were significantly lower compared to magnesium chloride hexahydrate (*p* < 0.05). Results for magnesium bisglycinate (66.66%) (magnesium amino acid chelate ALBION^®^) and magnesium oxide (59.76%) were significantly higher compared to models in which magnesium lactate dihydrate (*p* < 0.01), magnesium citrate (*p* < 0.01), and magnesium L-pidolate (*p* < 0.001) were used and also to the bioavailability of Mg only from the diet (*p* < 0.001). Moreover, results for magnesium lactate dihydrate and magnesium citrate were significantly lower compared to models in which magnesium chloride hexahydrate was used (*p* < 0.001).

Finally, statistically significantly higher results were demonstrated in experiments using the chemical form of magnesium chloride hexahydrate compared to magnesium L-pidolate and magnesium citrate (*p* < 0.001) and compared to diet (*p* < 0.001). Between the remaining chemical forms of Mg, for which the averaged results ranged from 57.12 to 66.66% no significant differences were determined. The results presented in Table 4 have shown that only in the case of magnesium oxide has no influence of the type of the diet been demonstrated. For other chemical forms of this macroelement, significant differences in the values of the bioavailability under the influence of various types of diets were shown.

With the addition of dietary supplements or medicinal products, it can be observed that the factors influencing the value of Mg bioavailability are the nutritional composition of the diets used in the study and the chemical form of Mg in which this element occurs in particular supplements or medicines. The results presented in Table 4 have shown that in the present study, the highest average bioavailability of Mg was determined for magnesium chloride hexahydrate, whereas the lowest was for magnesium L-pidolate. Based on the obtained results, it was also proved that the bioavailability of Mg was higher in all models in which dietary supplements or medicinal products were mixed with diets than from the sole diet only.

### 3.3. Influence of the Pharmaceutical Form on the Bioavailability of Mg

Dietary supplements and medicinal products containing Mg compounds used in the study were available in four pharmaceutical forms: coated tablets, sachets, capsules and tablets. Analyzing the effect of the pharmaceutical form of particular products used in the present study on the bioavailability of Mg from such systems, it was found that the highest value of this parameter was obtained in the case of the capsule form (62.77%), while the lowest in the models using dietary supplements in the form of sachets (53.86%). The results were presented in Table 6. Significantly higher results were determined in experiments using coated tablets compared to sachets (*p* < 0.001). Significant higher values of the bioavailability of Mg were also observed in experiments using capsules, compared to tablets (*p* < 0.01) and sachets (*p* < 0.001), as well as in comparison to the diet only (*p* < 0.001). Results between tablets and diet were also significant (*p* < 0.001). No statistically significant differences were observed between tablets and coated tablets.

## 4. Discussion

Mg is a key mineral that acts as a cofactor in over 300 biochemical reactions that are essential for maintaining the homeostasis of the body. The absorption of nutrients, including Mg, is undoubtedly influenced by dietary factors. They depend on the diet and include, among others, the chemical form of the dietary components, the dietary matrix, interactions between particular compounds, and methods of food preparation and processing. However, the physiological state of the host is also an important element. For example, reduced secretion of hydrochloric acid, gastric acid, and/or intrinsic factor, together with changes in intestinal mucosal permeability, are examples of intestinal factors that can significantly affect the absorption of some nutrients, but which are often ignored when establishing dietary requirements. Other factors that are worth considering include age, gender, physiological state, and the presence of chronic or inflammatory diseases [31]. In this study, it was shown that the bioavailability of Mg from the studied diets was within the range of 48.74–52.51%, and was lower in comparison to results for dietary supplements and medicinal products studied in the current experiments. The highest bioavailability of Mg was obtained from the standard diet (52.51%), and the lowest from the high-fiber diet (48.74%). It was proved that the bioavailability of Mg from pharmaceutical products may be higher than from a normal, mixed diet; therefore, Mg supplementation can be very beneficial, not only in terms of Mg quantities consumed, but also regarding the bioaccessibility of this ion. In the present study, it was observed that the factors influencing the bioavailability of Mg are the dietary matrix and the chemical form of Mg in which this element is present in pharmaceutical formulations. Commercially available preparations may contain Mg in the form of various inorganic compounds such as magnesium carbonate, oxide, sulfate, chloride, or nitrate. Organic Mg compounds used in the production process of dietary supplements and the few medicinal products available on the market include citrate, ascorbate, aspartate, gluconate, hydrogen aspartate, or lactate. According to the research of Jabłecka et al., the bioavailability of Mg from inorganic compounds ranged from 10 to 16% [32]. This was not confirmed in the present study, as it was found that inorganic forms of Mg were characterized by higher values of bioavailability compared to organic forms. Of the all Mg compounds studied in the present experiments, the highest bioavailability was determined for inorganic salt–magnesium chloride hexahydrate, for which the mean value reached almost 70%. Mg hydroxide, a simple inorganic compound, was also proved to have high bioavailability of around 65%. Among organic Mg compounds, only magnesium bisglycinate showed high bioavailability at the level of 67%. In an in vivo study on the bioavailability of Mg from dietary supplements available on the American market, Firoz and Graber showed that chemical forms of Mg, such as lactate, aspartate, and chloride, were characterized by better bioavailability compared to magnesium oxide. Present research confirmed a higher bioavailability of Mg from magnesium chloride in comparison to magnesium oxide, but did not confirm a higher bioavailability from magnesium lactate compared to magnesium oxide [33]. However, our research confirmed the conclusions of Firoz and Garber, that a simple statement that organic forms of magnesium are better absorbed than inorganic forms is untrue, because the bioavailability process is influenced by many factors related not only to the pharmaceutical product itself but also to the food matrix and the body’s needs. The in vitro conditions of the current experiment make it impossible to consider the Mg status of the body, but they do allow for the assessment of the influence of the composition of the food matrix on the bioavailability value. Rylander, analyzing numerous reports, noticed that magnesium citrate is characterized by better bioavailability compared to other organic salts of this element. Therefore, he considered that this chemical form is the most suitable for magnesium supplementation [34]. In the present study, although the bioavailability of Mg from magnesium citrate on average reached almost 60%, there were Mg compounds, which have been much better absorbed. Also, comparative studies of magnesium bioavailability conducted by Walker et al., showed better bioavailability of this macrooelement from its organic compounds than in this case from magnesium oxide. Three chemical forms of this element were compared: amino acid chelate, citrate and magnesium oxide at a dose of 300 mg/day. After 60 days of supplementation, the highest degree of bioavailability was determined for the chemical form of magnesium citrate. In the case of magnesium oxide, the results of supplementation were at the placebo level [35]. In the current study, it was shown that the results of the bioavailability of Mg from its inorganic compounds were on average at the level of 59.76–68.37%, while from its organic forms, within the range 53.86–66.66%. Therefore, the conducted studies did not show the advantage of organic forms of magnesium over inorganic forms of this element in the context of its bioavailability from dietary supplements or medicines, but in general, the bioavailability of this element was very high, in comparison to other elements, like Cr, Fe or Se, which was also revealed in our previous studies [23,24]. In vivo, magnesium citrate supplementation led to a significant increase in urinary magnesium excretion over 24 h, whereas magnesium oxide supplementation had no such effect. Magnesium citrate resulted in higher plasma magnesium levels than magnesium oxide. Furthermore, magnesium citrate supplementation significantly increased plasma magnesium levels compared to baseline levels, which was not observed after magnesium oxide administration [36]. In turn, in a recently published study conducted among healthy women, after short-term supplementation, magnesium oxide showed better bioavailability compared to other magnesium supplements (Mg citrate, Mg carbonate) [37]. However, a systematic review of 14 studies on the bioavailability of dietary supplements containing magnesium found that inorganic forms are less bioavailable than organic forms, and the extent of absorption is dose-dependent. However, all dietary supplements containing Mg can maintain physiological levels in healthy young people without previous deficiency [6]. According to many authors, the solubility of magnesium salts is crucial for their bioavailability. It is suggested that the better a given magnesium compound dissolves, the faster and in greater quantities its ions are released and may be absorbed [38,39]. Consequently, the presence of a highly soluble organic salt in a pharmaceutical product may probably contribute to a greater release of Mg ions compared to inorganic salts, which theoretically should translate into higher bioavailability. However, in the present study, no significantly higher bioavailability of Mg was observed for products containing highly soluble organic forms of this element. The exception was magnesium bisglycinate, which showed a relatively high level of bioavailability. Considering the influence of the pharmaceutical form on the bioavailability of Mg, it was found that the highest value of this parameter was obtained in the case of the capsule form (62.77%), while the lowest in the models using dietary supplements in the form of sachets (53.86%). According to studies conducted by Puścion-Jakubik et al., the best results of the bioavailability of magnesium were obtained from preparations in the form of a powder to dissolve in water [40]. In the case of powder to dissolve in water, the results obtained by Marzec et al. were very similar, where the result for sachets was 69.65% [41]. The lowest values were determined for dietary supplements available in the form of capsules, effervescent tablets and tablets [40]. Siener et al., evaluating in vivo the bioavailability of Mg in the form of magnesium oxide, from various pharmaceutical forms, showed better bioavailability of this element from effervescent tablets compared to capsules [42]. In the present study, the obtained results indicated the lowest bioavailability of Mg from the pharmaceutical form in the form of sachets with a result of 53.86%. The obtained results are inconclusive, and more studies are needed to fully elucidate this issue. Summarizing the obtained results, it is not possible to indicate a universal preparation that would be the most effective means of supplementing the deficiencies of this element, regardless of the type of diet used. For each type of diet, the highest bioavailability of Mg was obtained from a different dietary supplement or medicinal product. However, some trends can be noted. In the case of the best bioavailable compound magnesium chloride hexahydrate, all differences between particular diets were significant. The highest results were obtained for the basic diet, which was characterized by high protein and low fiber content. The same was observed for the best absorbed organic form magnesium bisglycinate, and the differences were also significant. The positive effect of the basic diet may also be explained by the low Mg content in the diet, which favored increased bioavailability of the element due to differences in osmotic pressure on both sides of the membrane. For other organic compounds, like Mg lactate or citrate, the most beneficial results were obtained in experiments using a high-residue diet, which contained high amounts of dietary fiber. Although dietary fiber and associated substances like phytates, have mineral-binding capacities, and therefore may reduce their absorption, this effect was proved to be the strongest for Ca, Zn or Fe, and magnesium absorption seems to be less affected [43]. These results may indicate a significant influence of the composition of the diet and particular nutrients on the bioavailability of Mg; therefore, even within the same pharmaceutical product, significant differences may be observed. The conducted experiments revealed that preparations containing Mg in the form of chloride hexahydrate and the pharmaceutical formula of capsules were characterized by the highest bioavailability of magnesium among the models used in the studies, although not all differences were significant.

A pharmaceutical product containing magnesium in the form of chloride had the status of registration as a drug. Its medical indication is magnesium supplementation in cases of magnesium deficiency. It was revealed that, from all analyzed supplements and drugs in the study, this pharmaceutical product was the most beneficial regarding the value of Mg bioavailability. It is possible that the technology of the drug form exceeds the solutions used in dietary supplement production and allows for a better release of Mg from a particular pharmaceutical form. Therefore, the effectiveness of this product in reducing magnesium deficiency may be higher than other supplements assessed in this study. However, additional studies are needed to confirm such a preliminary observation fully.

In bioavailability studies, various in vitro digestion models are used. The model used in this study is a static model in which the digestion conditions are determined once (pH, enzymes, temperature), while dynamic digestion models such as TIM (In Vitro Digestive Tract Model) or SHIME (Simulator of the Microbial Ecosystem of the Human Intestine) take into account peristalsis, dynamic pH changes and controlled enzyme secretion. The digestion model used includes a gastric and intestinal stage, which is standard in most in vitro digestion models, for example, the INFOGEST model (static in vitro simulation of food digestion in the digestive tract), which is also a static model, uses two stages of digestion. In more advanced models, additional stages are also added, e.g., intestinal fermentation as in the SHIME model. The digestion model used in the study takes into account the use of ground food instead of an exact imitation of a real food bolus, similar to the TIM, SHIME or INFOGEST models and many others. The ESIN (Engineered Stomach and Small Intestine) model uses a meal chamber that allows meals to be delivered to the stomach as mixtures of realistically sized particles. There is no perfect in vitro representation of the human digestive system, as each model has its limitations and does not account for all aspects of real digestion [44,45].

It should be remembered that the in vitro method using dialysis membranes only mimics the probable mechanisms occurring during the absorption of nutrients, but is not able to fully represent the real interactions taking place in the body, which can only be assessed in in vivo experiments. However, based on the literature data, it seems that in vitro digestion model coupled with cellulose membranes, is one of the best in vitro methods, used to evaluate the bioavailability of various nutrients and non-nutrients. What is important, this method is cheap, simple, lacks of any ethical concerns and does not require any animal studies [17,19,46,47].

## 5. Conclusions

The results of the conducted studies indicate a complex effect of a food matrix on the bioavailability of Mg from various pharmaceutical products. Based on the studied models it was observed that the factors influencing bioavailability are the composition of the diet and the chemical form of Mg. It was shown that inorganic forms of Mg are characterized by higher bioavailability compared to organic forms. Among the organic Mg compounds, only magnesium bisglycinate showed high bioavailability at a level similar to magnesium chloride, which was characterized by the highest bioavailability from all compounds evaluated in the study. Moreover, it was found that a higher value of this parameter was obtained for capsules, while the lowest was in models using dietary supplements in the form of sachets.

It was revealed that the composition of diet significantly influenced the bioavailability of Mg from various pharmaceutical products. However, there is no single universal product or diet that would be the most effective or beneficial in Mg supplementation in all cases, and the best choice depends on the form of magnesium, the pharmaceutical form of the preparation and individual dietary conditions. The conducted studies revealed that pharmaceutical preparations containing the active substance in the form of magnesium chloride hexahydrate and in the pharmaceutical form of capsules, especially in combination with a basic diet, were characterized by the highest bioavailability of Mg.

## Figures and Tables

**Table 1:** Detailed characteristics of dietary supplements used in the study.

Mg				
Product No.	Chemical Form	Supplement Type	Pharmaceutical Form	Registration Form
1	magnesium hydroxide	vitamin–mineral	coated tablets	dietary supplement
2	magnesium oxide	vitamin–mineral	coated tablets	dietary supplement
3	magnesium oxide	vitamin-mineral	capsules	medicinal product
4	magnesium oxide	single-mineral	capsules	dietary supplement
5	magnesium oxide	vitamin-mineral	tablets	dietary supplement
6	magnesium oxide	single-mineral	capsules	dietary supplement
7	magnesium oxide	vitamin-mineral	tablets	dietary supplement
8	magnesium bisglycinate (chelate ALBION^®^-Balchem Corporation, Montvale, NJ, USA) amino acid magnesium)	single-mineral	capsules	dietary supplement
9	magnesium lactate dihydrate	single-mineral	coated tablets	medicinal product
10	magnesium citrate	single-mineral	coated tablets	dietary supplement
11	magnesium chloride hexahydrate	single-mineral	coated tablets	medicinal product
12	magnesium L-pidolate	single-mineral	sachets for dissolving	dietary supplement

**Table 2:** Selective parameters of the applied analytical determinations.

Parameter	Mg (ICP-OES)
Reference value (mg/kg)	752.3
Determined value (mg/kg)	816.4
	734.2
	808.1
	794.5
	783.7
	771.9
**Average**	**784.8**
SD	29.54
RSD (%)	3.76
Recovery (%)	104.3
LOD (μg/kg)	37.0
LOQ (μg/kg)	135.7

SD—standard deviation; RSD—relative standard deviation; LOD—limit of detection; LOQ—limit of quantification.

**Table 3:** Bioavailability of Mg from dietary supplements and medicines under the influence of various types of diets.

Dietary Supplement No.	Chemical Form	Diet	M	Me	Min	Max	IQR	SD	One-Way ANOVA		Tukey’s Post Hoc Test Results		
									F	*p*	Group 1	Group 2	*p*
Without (%)	-	Standard	52.51	52.23	44.99	60.22	5.23	4.50	3.21	>0.05	Standard	Basic	>0.05
		Basic	52.23	51.77	46.71	57.75	2.14	3.00			Standard	High-residue	>0.05
		High-residue	48.74	48.65	43.21	52.59	2.89	2.79			Basic	High-residue	>0.05
1	magnesium hydroxide	Standard	46.95	48.51	39.52	54.38	9.41	5.43	209.68	<0.001	Standard	Basic	<0.001
		Basic	74.19	74.15	71.11	75.82	0.78	1.36			Standard	High-residue	<0.001
		High-residue	74.20	74.23	72.98	75.03	1.30	0.72			Basic	High-residue	>0.05
2	magnesium oxide	Standard	55.95	55.39	50.56	61.05	5.20	3.43	48.55	<0.001	Standard	Basic	<0.001
		Basic	64.01	63.59	61.32	66.70	2.22	1.80			Standard	High-residue	<0.001
		High-residue	66.01	65.82	64.78	67.23	1.36	0.89			Basic	High-residue	>0.05
3	magnesium oxide	Standard	69.63	68.97	58.15	83.84	8.22	7.49	143.17	<0.001	Standard	Basic	<0.001
		Basic	40.68	40.76	39.51	41.52	0.78	0.66			Standard	High-residue	<0.001
		High-residue	35.85	35.44	31.06	40.65	1.58	2.54			Basic	High-residue	>0.05
4	magnesium oxide	Standard	69.29	69.88	67.09	71.23	3.30	1.77	0.44	>0.05	Standard	Basic	>0.05
		Basic	70.51	70.93	67.09	71.55	0.94	1.38			Standard	High-residue	>0.05
		High-residue	69.63	69.91	60.95	77.51	2.95	4.40			Basic	High-residue	>0.05
5	magnesium oxide	Standard	36.54	36.19	35.14	38.56	1.92	1.26	1263.55	<0.001	Standard	Basic	<0.001
		Basic	64.79	64.79	63.85	65.91	0.92	0.67			Standard	High-residue	<0.001
		High-residue	49.20	49.18	47.29	51.03	2.91	1.50			Basic	High-residue	<0.001
6	magnesium oxide	Standard	76.55	75.91	73.52	79.32	3.35	2.03	304.86	<0.001	Standard	Basic	<0.001
		Basic	61.54	62.03	59.12	63.11	1.88	1.45			Standard	High-residue	<0.001
		High-residue	59.60	60.19	57.56	61.04	1.65	1.18			Basic	High-residue	<0.05
7	magnesium oxide	Standard	42.06	41.05	38.11	46.01	4.45	2.75	489.50	<0.001	Standard	Basic	<0.001
		Basic	73.03	74.09	69.92	75.79	4.06	2.24			Standard	High-residue	<0.001
		High-residue	70.89	72.13	67.99	72.88	2.87	1.97			Basic	High-residue	>0.05
8	magnesium bisglycinate	Standard	65.15	65.72	58.71	72.93	6.49	4.69	7.49	<0.01	Standard	Basic	<0.01
		Basic	70.08	70.48	65.42	73.73	2.88	2.56			Standard	High-residue	>0.05
		High-residue	64.76	64.81	62.28	66.48	0.52	1.12			Basic	High-residue	<0.01
9	magnesium lactate dihydrate	Standard	53.50	51.54	46.74	64.62	5.36	5.70	11.02	<0.001	Standard	Basic	>0.05
		Basic	56.43	56.93	54.11	58.54	2.62	1.59			Standard	High-residue	<0.001
		High-residue	61.42	61.42	58.14	63.94	3.20	2.05			Basic	High-residue	<0.05
10	magnesium citrate	Standard	52.86	52.39	49.71	58.42	1.37	2.57	43.84	<0.001	Standard	Basic	<0.001
		Basic	57.92	58.49	55.59	59.61	2.02	1.46			Standard	High-residue	<0.001
		High-residue	61.80	62.23	58.78	64.09	2.89	1.91			Basic	High-residue	<0.01
11	magnesium chloride hexahydrate	Standard	60.65	60.89	58.77	62.14	1.82	1.12	250.98	<0.001	Standard	Basic	<0.001
		Basic	75.27	74.95	73.55	77.05	1.88	1.33			Standard	High-residue	<0.001
		High-residue	69.19	69.44	66.67	71.70	1.72	1.67			Basic	High-residue	<0.001
12	magnesium L-pidolate	Standard	52.95	54.75 59.79 48.63	45.77 55.03 42.29	57.27 61.33 57.89	7.24 3.23 4.49	4.31 2.37 4.64	10.90	<0.001	Standard	Basic	<0.05
		Basic	58.54								Standard	High-residue	>0.05
		High-residue	50.09								Basic	High-residue	<0.001

M—arithmetic mean; Me—median; Min—minimum; Max—maximum; IQR—quartile range; SD—standard deviation; F—results of the ANOVA; *p*—significance value.

**Table 4:** Bioavailability of Mg considering chemical form under the influence of various types of diets.

Chemical Form	Diet	M	Me	Min	Max	IQR	SD	One-Way ANOVA		Tukey’s Post Hoc Test Results		
								F	*p*	Group 1	Group 2	*p*
Magnesium hydroxide	Standard	46.95	48.51	39.52	54.38	9.41	5.43	209.68	>0.001	Standard	Basic	<0.001
	Basic	74.19	74.15	71.11	75.82	0.78	1.36			Standard	High-residue	<0.001
	High-residue	74.20	74.23	72.98	75.03	1.30	0.72			Basic	High-residue	>0.05
Magnesium oxide	Standard	58.34	60.38	35.14	83.84	30.10	15.42	1.68	>0.05	Standard	Basic	>0.05
	Basic	62.43	64.79	39.51	75.79	8.570	10.68			Standard	High-residue	>0.05
	High-residue	58.53	60.99	31.06	77.51	19.11	12.80			Basic	High-residue	>0.05
Magnesium bisglycinate	Standard	65.15	65.72	58.71	72.93	6.49	4.69	7.49	<0.01	Standard	Basic	<0.01
	Basic	70.08	70.48	65.42	73.73	2.88	2.56			Standard	High-residue	>0.05
	High-residue	64.76	64.81	62.28	66.48	0.52	1.12			Basic	High-residue	<0.01
Magnesium lactate dihydrate	Standard	53.50	51.54	46.74	64.62	5.36	5.70	11.02	<0.001	Standard	Basic	>0.05
	Basic	56.43	56.93	54.11	58.54	2.62	1.59			Standard	High-residue	<0.001
	High-residue	61.42	61.42	58.14	63.94	3.20	2.05			Basic	High-residue	<0.05
Magnesium Citrate	Standard	52.86	52.39	49.71	58.42	1.37	2.57	43.84	<0.001	Standard	Basic	<0.001
	Basic	57.92	58.49	55.59	59.61	2.02	1.46			Standard	High-residue	<0.001
	High-residue	61.80	62.23	58.78	64.09	2.89	1.91			Basic	High-residue	<0.01
Magnesium chloride hexahydrate	Standard	60.65	60.89	58.77	62.14	1.82	1.12	250.98	<0.001	Standard	Basic	<0.001
	Basic	75.27	74.95	73.55	77.05	1.88	1.33			Standard	High-residue	<0.001
	High-residue	69.19	69.44	66.67	71.70	1.72	1.67			Basic	High-residue	<0.001
Magnesium L-pidolate	Standard	52.95	54.75	45.77	57.27	7.24	4.31	10.90	<0.001	Standard	Basic	<0.05
	Basic	58.54	59.79	55.03	61.33	3.23	2.37			Standard	High-residue	>0.05
	High-residue	50.09	48.63	42.29	57.89	4.49	4.64			Basic	High-residue	<0.001

**Table 5:** Average bioavailability of Mg considering chemical form.

Chemical Form	M	Me	Min	Max	IQR	SD	Kruskal–Wallis ANOVA	
							H	*p*
Magnesium hydroxide (%)	65.11	73.62	39.52	75.82	23.55	13.46	81.30	<0.001
Magnesium oxide (%)	59.76	63.94	31.06	83.84	20.98	13.16		
Magnesium bisglycinate (%)	66.66	65.72	58.71	73.73	5.70	3.91		
Magnesium lactate dihydrate (%)	57.12	57.59	46.74	64.62	6.04	4.82		
Magnesium Citrate (%)	57.53	58.49	49.71	64.09	7.27	4.21		
Magnesium chloride hexahydrate (%)	68.37	69.44	58.77	77.05	12.88	6.26		
Magnesium L-pidolate (%)	53.86	55.41	42.29	61.33	8.66	5.18		
Diet (%)	51.16	51.13	43.21	60.22	4.54	3.80		

H—test statistic value for the ANOVA test.

**Table 6:** Average bioavailability of Mg considering pharmaceutical form.

Pharmaceutical Form	M	Me	Min	Max	IQR	SD	Kruskal–Wallis ANOVA	
							H	*p*
Diet without preparation (%)	51.16	51.13	43.21	60.22	4.54	3.80	53.99	<0.001
Coated tablets (%)	62.02	61.42	39.52	77.05	11.81	8.62		
Capsules (%)	62.77	65.83	31.06	83.84	10.91	12.25		
Tablets (%)	56.08	57.44	35.14	75.79	28.88	14.43		
Sachets (%)	53.86	55.41	42.29	61.33	8.66	5.18		

## Data Availability

Data are contained within the article or Supplementary Materials.

## Supplementary Materials

The following supporting information can be downloaded at: https://www.mdpi.com/article/10.3390/nu17050748/s1, Table S1: Composition of diets used in the study, Table S2: Selected nutritional parameters of diets used in the study, Table S3: Operating parameters in the ICP-OES method.
