# Supplementary material for: In Vitro Evaluation of Bioavailability of Mg from Daily Food Rations, Dietary Supplements and Medicinal Products from the Polish Market
Source: Nutrients. 2025 Feb 20;17(5):748. doi: 10.3390/nu17050748 (PMC11901550; doi:10.3390/nu17050748)
Supplement: Supplementary file 1 [file nutrients-17-00748-s001.zip › nutrients-3470658-supplementary.pdf]

SUPPLEMENTARY FILE

In Vitro Evaluation of Bioavailability of Mg from Daily Food Rations, Dietary Supplements and Medicinal Products from the Polish Market

Piotr Bawiec [1], Agnieszka Jaworowska [1], Jan Sawicki [2], Marcin Czop [3], Radosław Szalak [1,4] and Wojciech Koch [2,*]

1 Department of Food and Nutrition, Medical University of Lublin, 4a Chodźki Str., 20-093 Lublin, Poland; piotr.bawiec@wp.pl (P.B.); agnieszka.jaworowska@umlub.pl (A.J.)

2 Department of Analytical Chemistry, Medical University of Lublin, 4a Chodźki Str., 20-093 Lublin, Poland; jan.sawicki@umlub.pl (J.S.)

3 Department of Clinical Genetics, Medical University of Lublin, Radziwiłłowska 11 Str., 20-080 Lublin, Poland; marcin.czop@umlub.pl (M.C.)

4 Department of Animal Anatomy and Histology, Faculty of Veterinary Medicine, University of Life Sciences,

12 Akademicka St., 20-950 Lublin, Poland; radek.szalak@up.lublin.pl (R.S.)

* Correspondence: kochw@interia.pl; Tel.: +48-81-448-7142

Table S1. Composition of diets used in the study [23,24].

Type of the meal	Food product/meal	Amount (g/mL)
**Basic diet**		
Breakfast	cereal coffee with milk	250
	white bread	80
	cottage cheese	110
	Onion	10
	cucumber	30
	butter	10 g

Second breakfast	mixed bread (wheat-rye flour)	60
	chicken pate	130
	apple	150
	black tea infusion	250
Lunch	vegetable soup (including carrots, celery, parsley, cauliflower)	400 (vegetables – 100 g)
	poultry chop	150
	potatoes	300
	red cabbage salad	150
	grated strawberry compote	250 (strawberries -30 g)
Dinner	meatballs in sauce	120
	pasta	120
	salad (carrot, apple, mayonnaise)	100
	yeast cake with crumble	50
	black tea infusion with milk	250
**Standard diet**		
Breakfast	ham sausages	150
	mixed bread (wheat-rye flour)	90

	cocoa with milk	250
	mustard	20
Second breakfast	gouda cheese	60
	crispbread	30
	coffee infusion with milk	150
Lunch	tomato soup with pasta	400
	Potatoes	300
	grilled cod	200
	salad with sauerkraut	150
	compote	250
	coffee infusion with milk	150
	milk chocolate with nuts	30
Dinner	mixed bread (wheat-rye flour)	80
	sausages	110
	pickled cucumber	80
	black tea infusion	250
**High-residue diet**		
Breakfast	oatmeal in milk	oat flakes – 50 g

		milk – 350 g
	wholemeal bread	80
	cottage cheese	80
	jam	20
Second breakfast	mixed bread (wheat-rye flour)	80
	rennet cheese	40
	ham	440
	tomatoes	150
	banana	100
	black tea infusion	250
Lunch	beetroot soup	400 (beetroot – 30 g; vegetables: carrots, parsley, celery – 25 g)
	pork chop	150
	potatoes	300
	boiled vegetables (carrots with peas 1+1 with breadcrumbs and butter)	300
	apple	150
	strawberry compote	200
Dinner	mixed bread (wheat-rye flour)	80

	chicken ham	80
	red pepper	150
	butter	10 g
	coffee infusion with milk	150

Table S2. Selected nutritional parameters of diets used in the study [23,24].

**Parameter**	**Diet**		
	**Basic**	**Standard**	**High-residue**
Proteins (g)	116.8 (17.3% of E*)	153.9 (22.2% of E)	143.5 (18.5% of E)
Fats (g)	109.2 (36.4% of E)	129 (41.9% of E)	117.1 (34% of E)
Carbohydrates (g)	338 (46.3% of E)	272.2 (35.8% of E)	416 (47.5% of E)
Fiber (g)	28.6	24.6	50.2
Vitamin A (μg)	2960	500	4600
Vitamin C (μg)	80.6	54.8	304.7
Vitamin E (mg)	17.2	15.9	24.7
Calcium (mg)	568.7	1203	1465
Sodium (mg)	2370	4865	3290
Potassium (mg)	4877	5615	6837
Magnesium (mg)	361	562.9	693
Iron (mg)	14.3	17.2	21.7
Energy (kcal)	2699	2770	3099
Total weight (g)	2970	2750	3285

* Percentage of energy

Table S3. Operating parameters in the ICP-OES method.

Analytical line, reading time	Mg 285.213 nm, 3 s
Signal reading type	Radial, attenuated
Signal integration	3 pix
Plasma generator power	1300 W
Coolant gas flow rate	14 $L \cdot min^{-1}$
Auxiliary gas flow rate	0.5 $L \cdot min^{-1}$
Carrier gas flow rate	0.6 $L \cdot min^{-1}$
Sample flow rate	1.0 $L \cdot min^{-1}$
